# Arteriogenic hemorrhage of the anastomotic loop after choledochojejunostomy: case series and literature review

**DOI:** 10.1016/j.radcr.2026.01.074

**Published:** 2026-02-13

**Authors:** Rilong Chen, Jingzhan Huang, Jinyong Lin, Jianlong Zhang

**Affiliations:** Department of Hepatobiliary Surgery, Shenshan Medical Center, Sun Yat-sen Memorial Hospital of Sun Yat-sen University, No. 1, Zhanqian Heng'er Road, Dongchong Town, Chengqu District, Shanwei City, Guangdong Province 516600, China

**Keywords:** Choledochojejunostomy, Upper gastrointestinal bleeding, Hepatic artery embolization, Computed tomography angiography, Digital subtraction angiography, Case report

## Abstract

Arteriogenic hemorrhage of the anastomotic loop after choledochojejunostomy is a life-threatening, and complex postoperative complication, necessitating a comprehensive diagnostic approach integrating clinical manifestations, imaging findings, and results from endoscopy or angiography. We present 3 clinical cases: a 22-year-old male who developed hemorrhagic shock 28 days after laparoscopic choledochal cyst resection, due to an anomalous communication between the right hepatic artery stump and the choledochojejunostomy, and achieving hemostasis via transarterial embolization; a 71-year-old female with painless hematochezia and shock 1 month after laparoscopic radical resection of middle common bile duct cancer, due to a proper hepatic artery pseudoaneurysm, whose symptoms were effectively controlled after transarterial embolization; and a 44-year-old male with hematochezia on 11 days after laparoscopic choledochal cyst resection, who failed interventional management, requiring laparotomy for gastroduodenal artery stump ligation to achieve hemostasis. Key etiologies of this complication encompass anastomotic leakage and vascular erosion; Timely interventional interventions (eg, transarterial embolization, covered stent placement) serve as effective first-line management, whereas laparotomy acts as a critical salvage strategy for refractory cases failing interventional therapy.

## Introduction

Choledochojejunostomy is a widely used reconstructive procedure in the field of hepatopancreatobiliary surgery, aimed at restoring biliary drainage. It is widely applied in the treatment of various diseases, including pancreaticoduodenectomy, hilar cholangiocarcinoma resection, and congenital choledochal cyst [1]. Although this procedure plays a crucial role in improving patient prognosis and quality of life, postoperative complications remain one of the key concerns in clinical practice [2]. Among various complications, vasogenic hemorrhage of the anastomotic loop, especially arteriogenic hemorrhage, though rare, its potential severity cannot be ignored and may even be life-threatening [3].

The pathogenesis of arteriogenic hemorrhage of the anastomotic loop is complex and diverse, involving multiple aspects [4]. Direct injury to local blood vessels during surgical manipulation is one of the main immediate causes. In the process of reconstructing the continuity of the biliary tract and digestive tract, blood vessels at the anastomotic site may be damaged due to suture techniques, tissue tension, or electrocoagulation injury. In addition, anastomotic infection or local inflammatory reactions can lead to vascular wall erosion and the formation of pseudoaneurysms. Once these fragile structures rupture, they will trigger massive arteriogenic hemorrhage [5]. In certain cases, especially in patients with portal hypertension, although variceal bleeding is more common, abnormal angiogenesis or portal venous system abnormalities may also indirectly increase the risk of arteriogenic hemorrhage. For example, studies have indicated that afferent loop varices in patients with malignant tumors after choledochojejunostomy can be identified by computed tomography (CT), suggesting the complexity of vascular lesions in this region [3].

The clinical manifestations of arteriogenic hemorrhage are usually more acute and dangerous. Patients may present with hemodynamic instability, melena, hematemesis, and other symptoms [6]. Due to the deep location of the bleeding site, which may be obscured by blood clots or surrounding tissues, early diagnosis is full of challenges. Imaging examinations, such as contrast-enhanced computed tomography angiography (CTA) and digital subtraction angiography (DSA), play a crucial role in localizing the bleeding source and evaluating vascular lesions [7]. However, due to the particularity of the anastomotic location, endoscopic techniques such as single-balloon or double-balloon assisted enteroscopy are sometimes required to directly observe and intervene in the anastomotic region [3].

For the treatment of arteriogenic hemorrhage of the anastomotic loop after choledochojejunostomy, the importance of early diagnosis and timely intervention is emphasized. Interventional embolization has become a minimally invasive and effective therapeutic method. By embolizing the bleeding artery through transcatheter technology, hemorrhage can be quickly controlled [8]. For cases where interventional therapy fails or there is massive hemorrhage, surgical exploration remains necessary salvage measures [4]. In terms of prevention, a comprehensive assessment of the patient's vascular status and coagulation function should be performed preoperatively. Intraoperatively, delicate surgical techniques should be adopted to avoid vascular injury and ensure good blood supply to the anastomotic site [1].

This article reports the diagnosis and treatment of 3 patients with Arteriogenic hemorrhage of the anastomotic loop after choledochojejunostomy and reviews relevant literature, aiming to explore the etiological characteristics, diagnostic methods, and treatment strategies of this complication, and provide references for clinical practice.

## Case presentations

### Case 1

#### Chief complaints

A 22-year-old male presented with acute abdominal pain and hematemesis 28 days after laparoscopic choledochal cyst resection, cholecystectomy, and Roux-en-Y choledochojejunostomy.

#### History of present illness

The patient presented with upper abdominal discomfort accompanied by acid reflux then underwent surgical resection for a congenital choledochal cyst and was discharged with uneventful recovery. Twenty-eight days postoperatively, he developed sudden-onset abdominal pain accompanied by hematemesis. Laboratory tests showed a hemoglobin decrease from a baseline of 11.9 g/dL to 8.2 g/dL, with no elevation in bilirubin. Abdominal CT revealed no intra-abdominal hemorrhage.

#### Physical examination

The patient presented with hemorrhagic shock (vital signs: blood pressure 85/50 mmHg, heart rate 131 beats per min).

Anemic appearance; no abdominal tenderness or peritoneal signs.

#### Laboratory examinations

Hemoglobin:8.2 g/dL(baseline: 11.9 g/dL);

Bilirubin: within normal range;

Alanine aminotransferase (ALT): 1037 U/L, aspartate aminotransferase (AST): 610 U/L (postoperatively elevated). Total Bilirubin: 30.5 umol/L

#### Imaging examinations

**Fig. 1:**
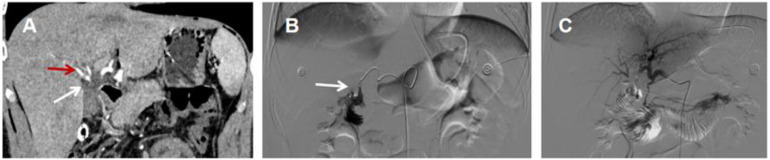
(A) Enhanced abdominal CT showing the right hepatic artery (red arrow) adjacent to the choledochojejunostomy anastomosis (white arrow); (B) Celiac arteriography confirming a rupture in the right hepatic artery with contrast leakage, directly communicating with the choledochojejunostomy anastomosis (white arrow) and (C) Repeat angiography after embolization of the right hepatic artery showing no contrast leakage, with loss of right hepatic blood flow and well-preserved left hepatic artery and left liver perfusion.

Contrast-enhanced abdominal CT: Adjacency between the right hepatic artery and the choledochojejunostomy site (Fig. 1A);

Celiac angiography: Rupture in the right hepatic artery with contrast leakage, directly communicating with the choledochojejunostomy site (Fig. 1B).

#### Treatment

Emergency transcatheter arterial embolization (TAE) of the right hepatic artery was performed using coils (Fig. 1C) .Postoperatively, the patient received supportive therapies including blood transfusion (4 units of packed red blood cells), fluid resuscitation, proton pump inhibitor (PPI) for acid suppression, and hepatoprotective agents (Magnesium Isoglycyrrhizinate+ polyene phosphatidylcholine).

#### Outcome and follow-up

Hemostasis was achieved immediately after TAE, and hemodynamic stability was restored within 24 hours. Liver enzymes gradually decreased and normalized after 1 year of continuous hepatoprotective therapy. No recurrent bleeding or long-term complications were observed during 2 years of follow-up.

### Case 2

#### Chief complaints

A 71-year-old female presented with painless hematochezia 1 month after laparoscopic radical resection of middle common bile duct cancer, Roux-en-Y choledochojejunostomy, and lymphadenectomy.

#### History of present illness

The patient with painless jaundice was diagnosed with middle common bile duct cancer and underwent percutaneous transhepatic cholangial drainage (PTCD) for jaundice relief prior to definitive surgery. She was discharged with a PTCD tube after uneventful postoperative recovery. One month postoperatively, she developed painless hematochezia, with hemoglobin decreasing from 8.8 g/dL to 5.6 g/dL. Then she developed hemorrhagic shock, altered consciousness, and decreased oxygen saturation (85% on room air).

#### Physical examination

Vital signs: Blood pressure 70/40 mmHg, heart rate 130 beats per min, oxygen saturation 85%;

Anemic appearance; no abdominal tenderness or peritoneal signs.

#### Laboratory examinations

Hemoglobin: 5.6 g/dL (baseline: 8.8 g/dL);

Total Bilirubin: 21.5 umol/L;

AST/ALT: Elevated postembolization (AST: 450 U/L, ALT: 380 U/L).

#### Imaging examinations

**Fig. 2:**
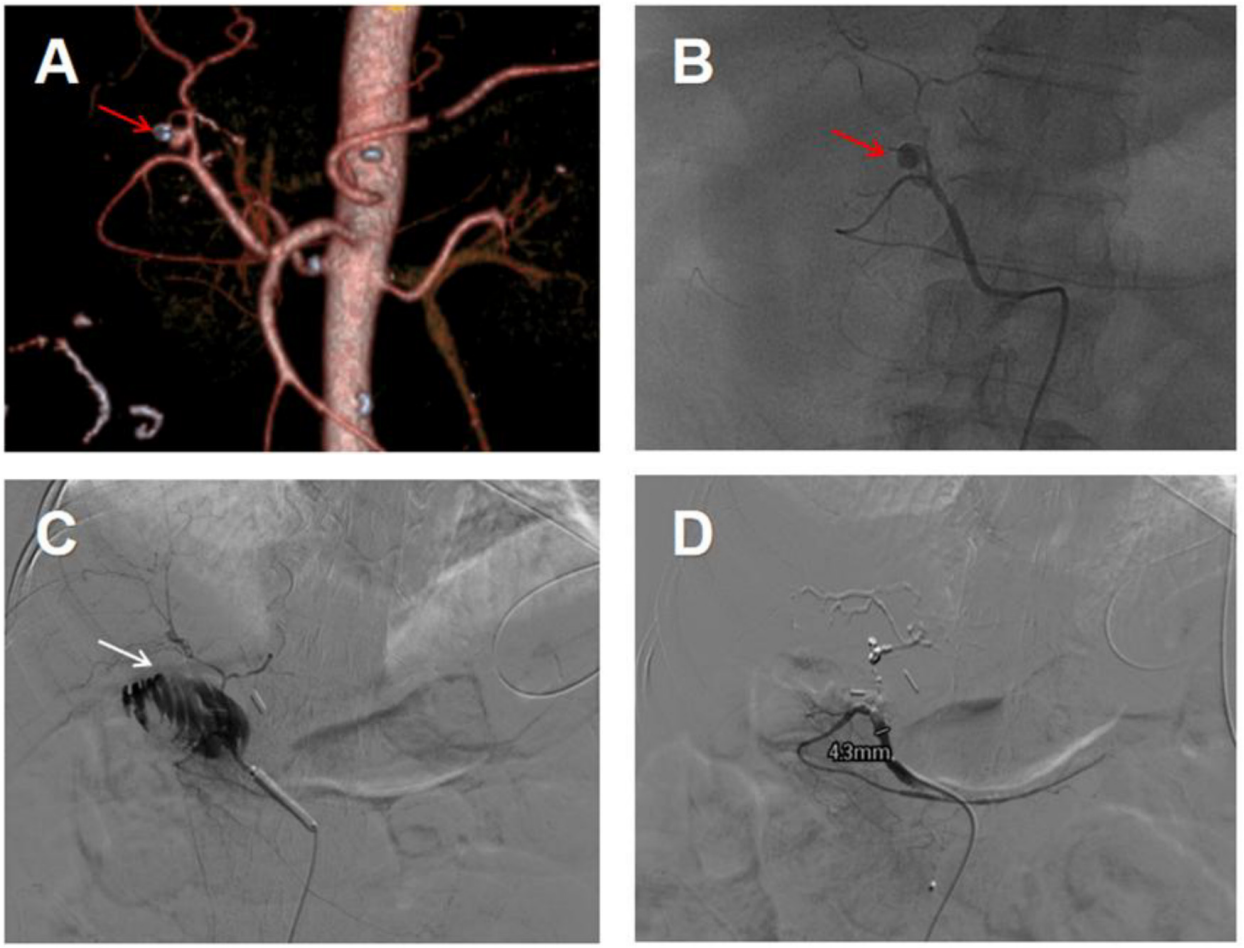
(A) Celiac artery CTA showing a pseudoaneurysm of the proper hepatic artery (red arrow); (B) Celiac arteriography confirming the proper hepatic artery pseudoaneurysm (red arrow); (C) Celiac arteriography confirming communication between the proper hepatic artery pseudoaneurysm and the intestine, with contrast leakage into the intestine (white arrow) and (D) Angiography after coil embolization of the proper hepatic artery showing no residual hepatic artery blood flow.

Contrast-enhanced CT + CTA: Proper hepatic artery pseudoaneurysm (Fig. 2A);

Celiac angiography: Pseudoaneurysm of the proper hepatic artery with contrast leakage into the intestinal tract (Figs. 2B and C).

#### Treatment

Emergency TAE was performed due to tortuosity of the aneurysm precluding covered stent placement. Coils were deployed to embolize the proper hepatic artery (Fig. 2D). Postoperatively, the patient received blood transfusion (6 units of packed red blood cells), fluid resuscitation, PPI, and hepatoprotective therapy.

#### Outcome and follow-up

**Fig. 3:**
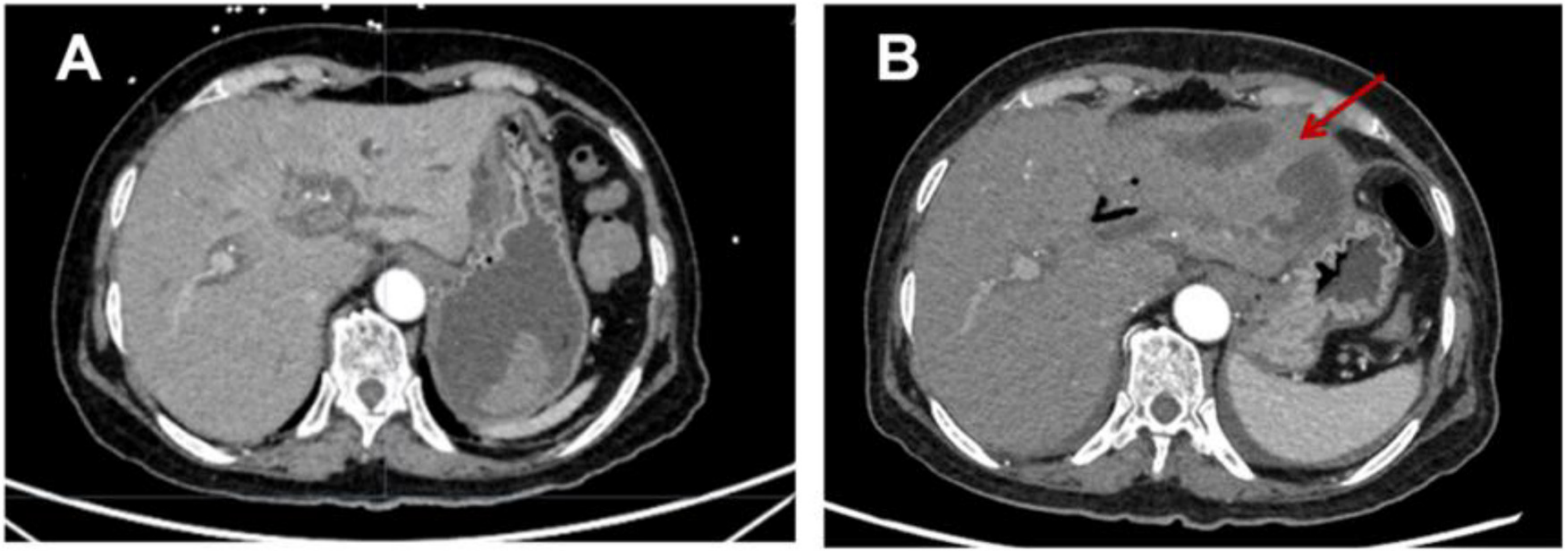
(A) Preoperative hepatic artery embolization and (B) Two weeks after hepatic artery embolization, arterial phase CT showing reduced liver parenchymal density, left hepatic softening, and a liquefied cavity (red arrow).

Shock resolved within 48 hours, but the patient developed hypertension (160/100 mmHg), fever (38.5°C), and right upper abdominal pain. Liver enzymes peaked at 1 week postoperatively (AST: 648 U/L, ALT: 572 U/L) and gradually normalized with continued hepatoprotective therapy. Two weeks postembolization, contrast-enhanced CT revealed hepatic infarction and liver abscess formation (Fig. 3), which were managed with antibiotics and percutaneous drainage. No recurrent bleeding was observed during 1 year of follow-up.

### Case 3

#### Chief complaints

A 44-year-old male presented with palpitations, profuse sweating, hematemesis, and hematochezia 11 days after laparoscopic choledochal cyst resection, cholecystectomy, and Roux-en-Y choledochojejunostomy.

#### History of present illness

The patient with upper abdominal discomfort underwent surgical resection for a congenital choledochal cyst and had an uneventful early postoperative course. Eleven days postoperatively, he developed acute hematemesis and hematochezia, with hemoglobin decreasing from 12.5 g/dL to 7.8 g/dL. Contrast-enhanced CT showed hemobilia, and angiography confirmed a proper hepatic artery pseudoaneurysm.

#### Physical examination

Vital signs: Blood pressure 90/60 mmHg, heart rate 115 beats per min;

Pallor; epigastric tenderness without rebound.

#### Laboratory examinations

Hemoglobin: 7.8 g/dL (baseline: 12.5 g/dL);

Bilirubin: within normal range.

#### Imaging examinations

**Fig. 4:**
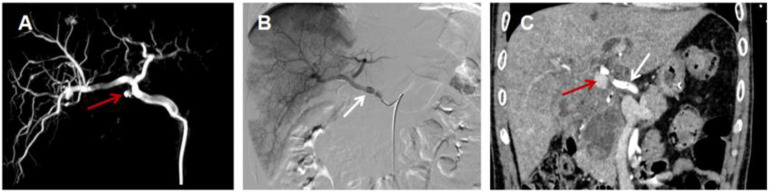
(A) Celiac arteriography showing a proper hepatic artery pseudoaneurysm (red arrow); (B) Postoperative angiography after proper hepatic artery covered stent placement (white arrow) showing normal arterial course, patent distal flow, and no contrast leakage and (C) Abdominal CTA after covered stent placement (white arrow) showing contrast leakage (red arrow).

Contrast-enhanced abdominal CT: hemobilia with no intra-abdominal hemorrhage;

Celiac angiography: contrast extravasation from the proper hepatic artery (Fig. 4A);

Poststent CTA: persistent contrast leakage (Fig. 4C).

#### Treatment

Initial treatment included covered stent placement and balloon angioplasty of the proper hepatic artery (Fig. 4B). However, rebleeding occurred 2 days postoperatively, with hemoglobin dropping to 6.5 g/dL. Laparotomy was performed, identifying bleeding from the gastroduodenal artery stump, which was controlled with suture ligation. Postoperatively, the patient received blood transfusion (5 units of packed red blood cells), fluid resuscitation, PPI, and prokinetic therapy (mosapride).

#### Outcome and follow-up

Hemostasis was achieved definitively after surgery. The patient developed upper abdominal distension 2 months postoperatively, which resolved with continued prokinetic therapy. No recurrent bleeding or other complications were observed during 18 months of follow-up.

## Discussion

This study reports 3 cases of upper gastrointestinal bleeding after choledochojejunostomy, all were arteriogenic hemorrhage. The vasogenic etiology was confirmed by imaging examinations in all cases, and their clinical characteristics, diagnosis, and treatment processes provide important references for the individualized management of bleeding after choledochojejunostomy (Comparison see Table 1).

**Table 1:** Comparison of clinical characteristics among 3 cases of arteriogenic hemorrhage after choledochojejunostomy.

Clinical parameters	Case 1	Case 2	Case 3
Age/Gender	22 years/Male	71 years/Female	44 years/Male
Underlying disease	Congenital choledochal cyst	Middle common bile duct cancer	Congenital choledochal cyst
Initial surgical procedure	Laparoscopic choledochal cyst resection + cholecystectomy + Roux-en-Y choledochojejunostomy	Percutaneous transhepatic cholangial drainage (PTCD) + laparoscopic radical cholangiocarcinoma resection + choledochojejunostomy + lymphadenectomy	Laparoscopic choledochal cyst resection + cholecystectomy + Roux-en-Y choledochojejunostomy
Postoperative bleeding time	28 days after surgery	1 month after surgery	11 days after surgery
Main clinical manifestations	Acute abdominal pain, hematemesis, hemorrhagic shock (BP: 85/50 mmHg, HR: 131 bpm)	Painless hematochezia, hemorrhagic shock, altered consciousness, decreased oxygen saturation (85% on room air) (BP: 70/40 mmHg, HR: 130 bpm)	Palpitations, profuse sweating, hematemesis, hematochezia (BP: 90/60 mmHg, HR: 115 bpm)
Baseline hemoglobin/Hemoglobin at bleeding	11.9 g/dL / 8.2 g/dL	8.8 g/dL / 5.6 g/dL	12.5 g/dL / 7.8 g/dL (reached 6.5 g/dL after rebleeding)
Total bilirubin	30.5 μmol/L	21.5 μmol/L	Within normal range
Bleeding site/Etiology	Anomalous communication between the right hepatic artery stump and choledochojejunostomy site	Proper hepatic artery pseudoaneurysm	Proper hepatic artery pseudoaneurysm; active bleeding from gastroduodenal artery stump (secondarily identified)
Key diagnostic methods	Contrast-enhanced abdominal CT, celiac angiography	Contrast-enhanced upper abdominal CT + computed tomography angiography (CTA), celiac angiography	Contrast-enhanced abdominal CT, arteriography, abdominal CTA, exploratory laparotomy
Core treatment regimen	Emergency transcatheter arterial embolization (TAE) of the right hepatic artery with coils; supportive therapy (4 units of packed red blood cells, PPI, hepatoprotective agents: Magnesium Isoglycyrrhizinate + polyene phosphatidylcholine)	Emergency TAE of the proper hepatic artery with coils; supportive therapy (6 units of packed red blood cells, PPI, hepatoprotective agents)	Initial: Hepatic artery covered stent placement + balloon angioplasty; Secondary: Exploratory laparotomy with suture ligation of gastroduodenal artery stump; supportive therapy (5 units of packed red blood cells, PPI, prokinetic agent: mosapride)
Postoperative complications	Elevated liver enzymes (peak ALT: 1037 U/L, peak AST: 610 U/L)	Hypertension (160/100 mmHg), fever (38.5°C), right upper abdominal pain, elevated liver enzymes (peak AST: 648 U/L, peak ALT: 572 U/L), hepatic infarction, liver abscess	Epigastric distension
Follow-up duration/Outcomes	2 years; liver function normalized after 1 year of hepatoprotective therapy, no recurrent bleeding	1 year; symptoms gradually controlled with hepatoprotective therapy and treatment for liver abscess, no recurrent bleeding	18 months; epigastric distension relieved with prokinetic therapy, no recurrent bleeding

**I. Correlative analysis of bleeding time and etiology**

The bleeding times of the 3 patients were 11 days, 28 days, and 1 month after surgery, respectively, which are all consistent with the definition of "delayed hemorrhage" (72 hours to 3 months postoperatively) [9], and the etiologies were all attributed to arterial injury. Specifically, Case 1 was due to an anomalous communication between the right hepatic artery stump and the anastomotic site, while Cases 2 and 3 were caused by proper hepatic artery pseudoaneurysms.

The formation of pseudoaneurysms may be associated with the following factors: 1) Occult injury to the vascular wall by energy devices during laparoscopic surgery, such as thermal injury to the arterial wall induced by ultrasonic scalpels or electrocautery hooks; during skeletonized lymphadenectomy, key vessels including the gastroduodenal artery and proper hepatic artery were not double-ligated or reinforced with sutures, leading to gradual pseudoaneurysm formation postoperatively; 2) Perianastomotic infection or bile leakage-induced vasculitis that erodes the arterial wall, resulting in arterio-biliary fistula. This also explains why none of the 3 cases had intra-abdominal hematoma, with bleeding confined to the biliary system.

**II. Selection of diagnostic methods and efficacy evaluation**

The bleeding sites were confirmed by computed tomography angiography (CTA) or digital subtraction angiography (DSA) in all 3 patients, confirming the core value of imaging examinations in vasogenic bleeding. As an initial screening tool, CTA can quickly localize the bleeding range (eg, proper hepatic artery pseudoaneurysm in Case 2, intraductal hematoma in Case 3), but its sensitivity for minor vascular leakage is lower than that of DSA. In contrast, DSA not only allows superselective angiography of the surgical field vessels to directly visualize contrast extravasation (Case 3) but also enables simultaneous interventional treatment [10].

Notably, none of the 3 cases presented with the typical "Quincke's triad" (right upper abdominal pain, bleeding, jaundice), suggesting that the clinical manifestations of biliary bleeding after choledochojejunostomy may be atypical [11]. Proactive imaging screening is required to avoid delayed diagnosis due to overreliance on typical symptoms.

**III. Individualized Selection of Treatment Strategies and Challenges**

The treatment processes of the 3 patients reflected the "intervention-first, surgery-as-salvage" principle, but also exposed the limitations of different treatment methods:

### Transcatheter arterial embolization

Hemostasis was achieved with hepatic artery embolization in Cases 1 and 2, but both patients developed liver function impairment, which were considered related to hepatic parenchymal ischemia induced by embolization. The incidence of hepatic ischemia after embolization is approximately 5%-10%, and the risk of liver failure should be vigilantly monitored. Hepatic intrahepatic bile duct ischemia can cause intrahepatic bile duct necrosis, biloma, and liver abscess formation, so the selection of embolized vessels must be cautious [12]. We observed that in Case 1, immediately after embolization of the right hepatic artery, the patient developed liver function impairment, but the left hepatic artery supply area provided intrahepatic collateral circulation to perfuse the ischemic right hepatic region, leading to gradual recovery of liver function. In Case 2, after embolization of the proper hepatic artery, hepatic blood supply relied solely on portal venous flow, The patient had transiently developed liver function impairment but also showed gradual recovery, with no liver failure during long-term follow-up. Therefore, despite not being the optimal choice, hepatic artery embolization can be a life-saving treatment option in emergency situations to rescue patients, with the expectation that long-term hepatic collateral blood flow will improve liver function.

### Covered stent placement

Compared with simple coil embolization, covered stents can preserve normal blood supply to the corresponding organ, reduce the risk of organ ischemia and necrosis, and minimize complications such as postembolization syndrome, liver abscess, and liver failure. However, in Case 3, initial covered stent placement for pseudoaneurysm occlusion failed, with recurrent bleeding 2 days postoperatively, suggesting that stent migration or vascular anatomical abnormalities (eg, tortuosity, multiple branches) may lead to treatment failure. Studies have shown that covered stents for hemostasis are generally suitable for vessels with an internal diameter >6 mm, and not for tortuous, stenotic, or small vessels [13].

### Laparotomy

Case 3 was converted to laparotomy for suture ligation of the gastroduodenal artery stump after failed interventional therapy, achieving definitive hemostasis. This indicates the limitations of vascular intervention in identifying bleeding sites; In cases where active nonsurgical or vascular interventional treatments fail to control bleeding, prompt laparotomy should be performed without delay [14].

**IV. Prognostic factors and long-term management**

All 3 patients developed postoperative liver dysfunction, which resolved with hepatoprotective therapy. Case 2 developed hepatic liquefactive lesions, suggesting that ischemic liver injury is a common complication of vascular interventional therapy. Monitoring of liver enzymes and coagulation function closely is necessary postoperative. Rational use of proton pump inhibitors and gastric mucosal protectants is recommended to prevent and treat postoperative stress ulcer bleeding. Long-term hepatoprotective agents should be administered to control liver damage, and appropriate prokinetic agents and intestinal flora regulators can be used to alleviate gastrointestinal dysfunction.

## Conclusion

Based on the cases in this study and literature review, the diagnosis and treatment of arteriogenic hemorrhage after choledochojejunostomy require attention to the following points: 1) Excessive thermal injury to the vascular wall should be avoided during laparoscopic surgery, and precise anatomical techniques should be adopted especially when handling hepatic artery branches; 2) Delayed bleeding should be vigilantly monitored 1-3 months postoperatively, with regular checks of hemoglobin and liver enzymes; 3) For suspected vasogenic bleeding, the "CTA screening + DSA treatment" process should be initiated as early as possible to avoid unnecessary delays from gastroscopy. Interventional therapy should prioritize revascularization and organ function-preserving embolization; if these goals cannot be achieved, hepatic vessel-sacrificing embolization can be selected to save the patient's life, with the expectation that long-term hepatic collateral blood flow will improve liver function. Timely laparotomy is indicated for failed interventional therapy; 4) Long-term follow-up should focus on liver and gastrointestinal function, with prompt intervention when necessary. Future research should further explore the association between the local anastomotic microenvironment (eg, bile components, flora) and vascular injury, providing new targets for preventing postoperative bleeding.

## Patient consent

Written informed consent was obtained from each patient (or their legally authorized representatives, where applicable) for the publication of this case report and any accompanying diagnostic images (including computed tomography angiography, celiac arteriography).

## Ethical Statement

All procedures involving human participants were conducted in accordance with the Declaration of Helsinki (2013 revision). Informed consent was obtained from all patients (or their legal representatives) for the collection, use, and publication of clinical data, imaging materials, and case details. Patient identifiers have been fully anonymized to protect privacy.
